# *Hydrostachys flabellifera* (Hydrostachyaceae), a new species from Madagascar

**DOI:** 10.3897/phytokeys.167.58538

**Published:** 2020-11-20

**Authors:** Zhun Xu, Jing Tian, Solo Hery Jean Victor Rapanarivo, Rokiman Letsara, Rivontsoa A. Rakotonasolo, Guy E. Onjalalaina, Guang-Wan Hu, Qing-Feng Wang

**Affiliations:** 1 Key Laboratory of Plant Germplasm Enhancement and Specialty Agriculture, Wuhan Botanical Garden, Chinese Academy of Sciences, Wuhan 430074, China; 2 Sino-Africa Joint Research Center, Chinese Academy of Sciences, Wuhan 430074, China; 3 University of Chinese Academy of Sciences, Beijing 100049, China; 4 Département Flore, Parc Botanique et Zoologique de Tsimbazaza, Antananarivo 101, Madagascar; 5 Germplasm Bank of Wild Species, Kunming Institute of Botany, Chinese Academy of Sciences, Kunming 650201, China

**Keywords:** Aquatic plants, Cornales, endemic, new taxa, taxonomy

## Abstract

*Hydrostachys flabellifera*, a new species of Hydrostachyaceae found in a stream in Manandriana, Madagascar, is described and illustrated herein. It is similar to *H. verruculosa* and *H. laciniata* in morphology, but can be distinguished from them by its leaves with sparsely arranged, flabelliform and palmately parted emergences, obvious rachis and the pattern of segments arranged on the male bracts. Molecular phylogenetic analysis of the nuclear ribosomal internal transcribed spacer (ITS) dataset provides a robust support for it as a new species as well.

## Introduction

*Hydrostachys*Thouars (1806: 2) is the sole genus in the family Hydrostachyaceae (Tul.) Engler (1894: 136) with about 22 known species. Fourteen of them are endemic to Madagascar (Phillipson et al. 2018) and the remaining species are native to southern and tropical Africa. *Hydrostachys* has been used in traditional medicine and probably could be a potential candidate for use in chemotherapy to fight against cancer (Ranarijaona et al. 2014). The plants of *Hydrostachys*, which are aquatic herbs living in fast-moving streams or rivers, are well adapted to turbulent aquatic environments with their roots and discoidal rhizome adhering to the rocks. *Hydrostachys* are annual or perennial, submerged or partially submerged in the rainy season, flowering in the dry season. Their simple or pinnate leaves emerge from the rhizome, and petiole, rachis and subdivisions are often covered with diverse forms of emergences, including verrucae, scales and lobules (modified leaf blade lobes), which give the plant the appearance of a fern or lycopodium. *Hydrostachys* are dioecious or seldom monoecious, with highly reduced and unisexual flowers borne on the spike, the spikes usually emerging from the rhizome, sepals and petals are absent; the fruit is a capsule with numerous tiny seeds (Perrier 1952; Cusset 1973; Stannard 1997; Verdcourt 1986; Erbar and Leins 2004).

*Hydrostachys* are highly modified aquatic plants and the taxonomic placement of this enigmatic genus has confounded botanists for two hundred years. Due to their similar habitat and highly modified morphological characters, the genus was once placed in Podostemaceae (Tulasne 1849). However, this placement was rejected thanks to evidence from embryology, inflorescence morphology (Jäger-Zürn 1965; Rauh and Jäger-Zürn 1966) and biochemistry (Scogin 1992). Phylogenetic studies showed unstable placements, based on different DNA markers and taxon sampling (Les et al. 1997; Soltis et al. 2000; Burleigh et al. 2009). Currently, Hydrostachyaceae is treated as a distinctive family in Cornales with a phylogenetic long branch in most studies (Albach et al. 2001; Xiang et al. 2002; Fan and Xiang 2003; Xiang et al. 2011; Fu et al. 2019). Despite the uncertain placement at the order level, the inter-species identification is much clearer.

During a field investigation in Madagascar in 2017, a *Hydrostachys* population was found in Manandriana which appeared similar to *H. verruculosa* A. Juss. (1837: tab. 91) and *H. laciniata*Warming (1899: 152). However, after carefully comparing the collection with all available specimens of *Hydrostachys* and consulting relevant literature (Perrier 1952; Cusset 1973), we observed that its leaf structure is different from that of all known species in this genus and that this plant is wholly new to science. Hence, we describe it herein as a new species.

## Materials and methods

The description of the new species is based on field notes and observations of field pictures, dried specimens and FAA-fixed (formalin/acetic acid/alcohol) materials. Specimens were collected from Manandriana, Madagascar (20°14'S, 47°06'E) and deposited at the herbaria of Parc Botanique et Zoologique de Tsimbazaza (TAN) and Wuhan Botanical Garden, Chinese Academy of Sciences (HIB). Some leaves and spikes were fixed and conserved in formalin/acetic acid/alcohol (FAA) fixatives. Detailed characteristics of the bracts and emergences were observed and measured on the fixed materials by using a stereomicroscope (Nikon Stereo Microscope SMZ25). Terminology was referenced in several books and literature (Perrier 1952; Cusset 1973; Verdcourt 1986; Simpson 2010; Beentje and Williamson 2016). The herbarium abbreviations follow Index Herbariorum (http://sweetgum.nybg.org/science/ih/). Physical specimens of *Hydrostachys*, deposited at BM, E, K and TAN, were examined. High-resolution digital specimen images from B, BNRH, BR, GH, MA, P and US were checked via JSTOR Global Plants (https://plants.jstor.org) and GBIF (https://www.gbif.org). The distribution map was produced by QGIS3 (available from: https://qgis.org/).

The nuclear ribosomal ITS was used as the DNA marker, with 13 samples included in the phylogenetic analysis. All *Hydrostachys* sequences were newly generated, while three taxa from *Nyssa* and one from *Triphyophyllum* were treated as outgroups. GenBank accession numbers are available in Table 1. Genomic DNA was extracted from dry specimens using Mag-MK Plant Genomic DNA extraction kits (Sangon Biotech, Shanghai). Primers for polymerase chain reactions (PCR) were referred to White et al. (1990). PCR products were sequenced by Sangon Biotech using the 3730xl DNA Analyzer and Geneious v.11.1.5 (available from: http://www.geneious.com/) was used for DNA assembling and manually editing. The dataset was aligned by MAFFT v.7.294 (Katoh and Standley 2013), then trimmed by trimAl v.1.2 (Capella-Gutierrez et al. 2009). The Maximum Likelihood tree was inferred using IQ-TREE v.2.0.6 (Minh et al. 2020) with default parameters and ultrafast bootstrap approximation was assessed with 1000 replicates. The consensus tree was visualised and annotated by ggtree v.2.2.1 (Yu et al. 2017). Dataset, scripts and command lines in the phylogenetic analysis are available in Github (https://github.com/xuzhun1008/Hydrostachys_flabellifera_paper.git).

**Table 1.** Taxa included in the phylogenetic analysis.

Species	Locality	Voucher	GenBank accession number
*Triphyophyllum peltatum* (Hutch. & Dalziel) Airy Shaw	–	TR121	HM204913
*Nyssa sylvatica* Marshall	–	zhangcq0088	JF977171
*Nyssa wenshanensis* Fang & Soong	China, Yunnan	S2007041304	JQ280761
*Nyssa javanica* Wangerin	–	S2007040302	JQ280777
*Hydrostachys multifida* A. Juss.	Madagascar, Boeny, Betsiboka	SAJIT3437	MW233025
*Hydrostachys longifida* H. Perrier	Madagascar, Analamanga, Antananarivo-Atsimondrano	SAJIT3442	MW233026
*Hydrostachys stolonifera* Baker	Madagascar, Vakinankaratra, Antanifotsy	SAJIT3446	MW233027
*Hydrostachys multifida* A. Juss.	Madagascar, Amoron'i Mania, Manandriana	SAJIT3453	MW233028
*Hydrostachys flabellifera* G.W. Hu, Zhun Xu & Q.F. Wang	Madagascar, Amoron'i Mania, Manandriana	SAJIT3462	MW233029
*Hydrostachys imbricata* A. Juss.	Madagascar, Vatovavy-Fitovinany, Ifanadiana	SAJIT3473	MW233030
*Hydrostachys multifida* A. Juss.	Madagascar, Vatovavy-Fitovinany, Ifanadiana	SAJIT3484	MW233031
Hydrostachys distichophylla var. hildebrandtii (Engl.) C. Cusset	Madagascar, Haute-Matsiatra, Iarintsena	SAJIT3490	MW233032
*Hydrostachys multifida* A. Juss.	Madagascar, Haute-Matsiatra, Ambalavao	SAJIT3498	MW233033

## Taxonomy

### Hydrostachys flabellifera

Taxon classificationPlantaeCornalesHydrostachyaceae

G.W. Hu, Zhun Xu & Q.F. Wang sp. nov.

FD8A4AE1-FC02-5E33-842F-B927217F738D

urn:lsid:ipni.org:names:77212953-1

Figs 1 , 2

#### Diagnosis.

*Hydrostachys flabellifera* is similar to *H. verruculosa* and *H. laciniata* in having simple leaves, but it can be easily distinguished from these species by short leaves, 3–12 cm long, the sparsely and spirally-arranged, flabelliform and palmately-parted emergences, the presence of a distinct and thin rachis between emergences and the pattern of segments arranged on the male bracts with acute apex.

**Figure 1.**
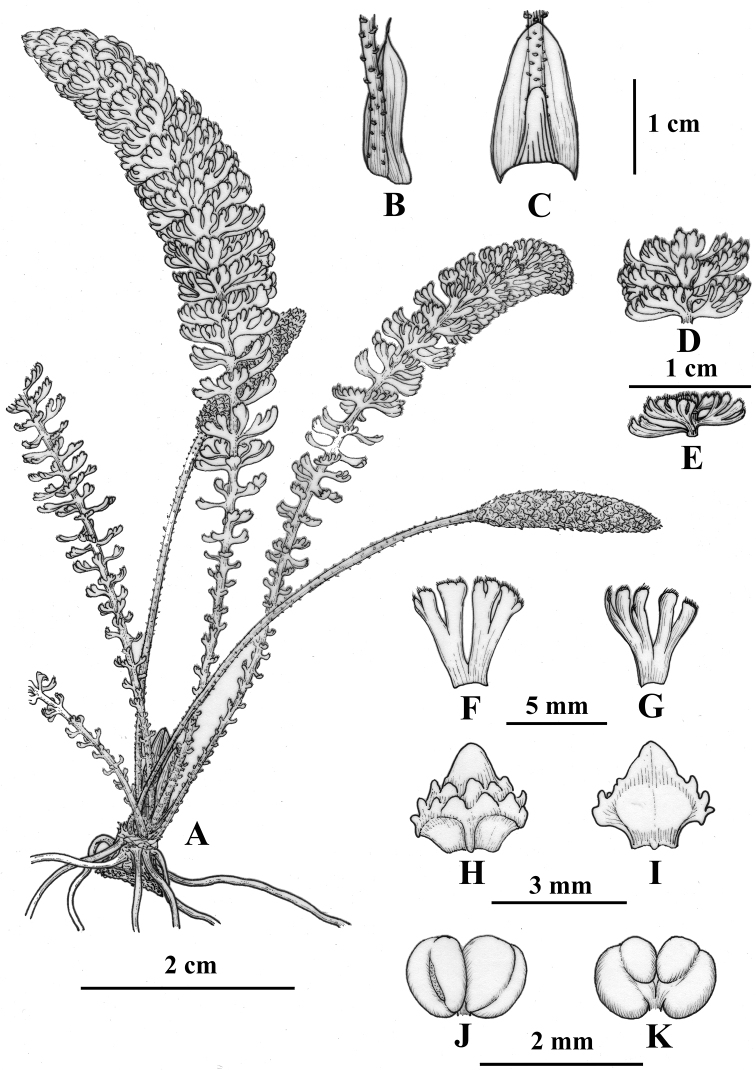
*Hydrostachys flabellifera* G.W. Hu, Zhun Xu & Q.F. Wang. **A** habit **B** lateral view of stipule and petiole base **C** ventral view of stipule and petiole base **D, E** part of the leaf **F** downside of a leaf emergence **G** upperside of a leaf emergence **H** dorsal view of male bract **I** ventral view of male bract **J, K** stamen. Drawn by Jing Tian.

#### Type.

Madagascar. Fianarantsoa Province: Amoron’i Mania Region, Manandriana District, elev. 1400 m, 20°14'S, 47°06'E, 20 September 2017, *Sino-Africa Joint Investigation Team (SAJIT)-003462* (holotype, HIB!, isotypes, HIB!, TAN!)

#### Description.

A hydrophyte herb. Rhizomes discoid, 3–8 mm in diameter; 7–12 leaves emerging from the rhizome. Leaves simple, 3–12 cm long, the upper part slightly curved when rising from water, the base enlarged with stipule; stipule ovate-elliptical, basal half dorsally attached on petiole, apex sometimes with a tail ca. 1.5 mm; centre bud enclosed by stipules of inner leaves; emergences spirally arranged on rachis and stretching out into loose layers, denser towards the apex of the leaf and gradually reduced to the base. Rachis obvious, 1–2 mm in diameter, white to light green. Petioles indistinct. Emergences (modified leaf blade lobes) 1–6 mm long, flabellate, basal ones reduced into scale-like, upper ones palmately parted, lobes cuneiform, secondly divided into rectangular to linear terminal lobes; the flat of emergences almost perpendicular to the axis; the apex of emergence slightly rolling up, ciliate at the end, cilia gathering into tufts after rising from water; emergences green to mauve at the pinnacle, the rest dark green. Male spikes 5.4–8.0 cm long, peduncles 4.7–6.2 cm long, covered with few small scale-like emergences. Bracts 1–2.2 mm × 1–2.2 mm, rhombic, dark green; segment I (the terminal segment) acute, flanked by 1–2 tiny lobules on each side; segments II (lobules at the dorsal side of bract) acute or obtuse, 2 rows, each row with 3–5 separated lobules, lobules ca. 0.3 mm high. Stamen sessile, anther oblate, with two divergent thecae dehiscing longitudinally. Female spike not found.

**Figure 2.**
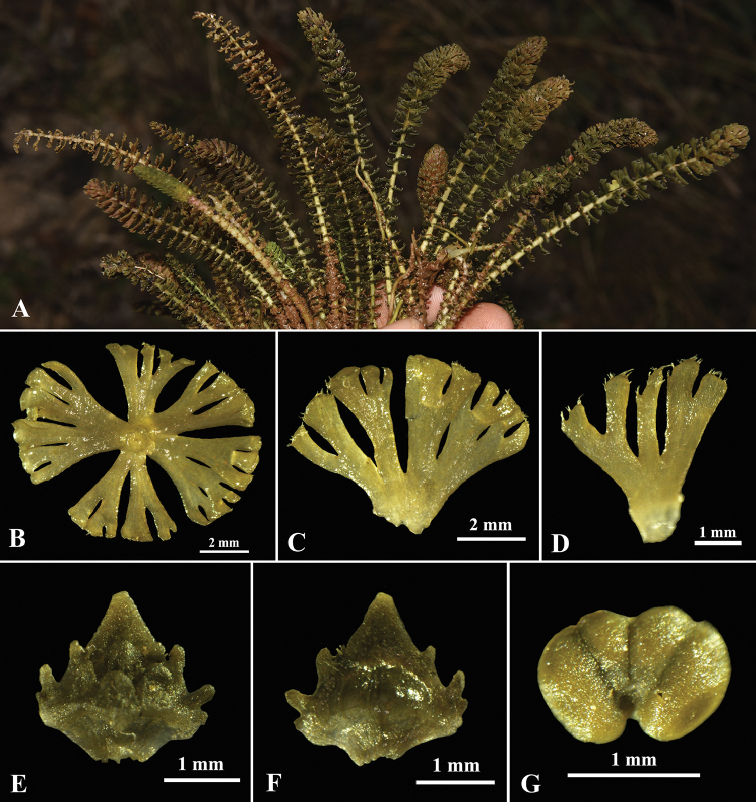
*Hydrostachys flabellifera* G.W. Hu, Zhun Xu & Q.F. Wang **A** habit **B** emergences on rachis, cross-section **C, D** emergences **E** dorsal view of male bract **F** ventral view of male bract **G** top view of stamen.

#### Etymology.

The epithet refers to the flabellate shape of emergences on leaves.

#### Distribution and ecology.

**Figure 3.**
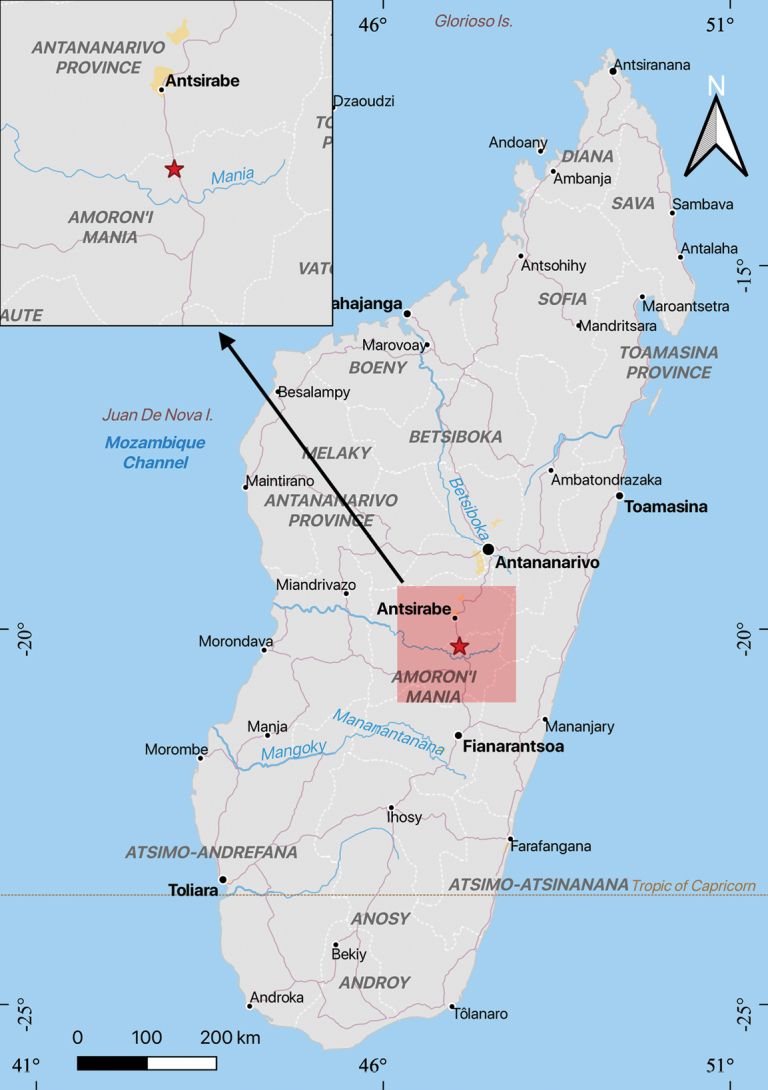
Distribution of *Hydrostachys flabellifera* G.W. Hu, Zhun Xu & Q.F. Wang.

Only one population was found on rocks in a stream in Manandriana, Madagascar (20°14'S, 47°06'E), at an elevation of 1400 m (Fig. 3).

#### Conservation status.

*Hydrostachys flabellifera* is currently only known from one location with a very small population. Additionally, all species of *Hydrostachys* are highly dependent on the moving aquatic environment which is threatened by water pollution, natural system modifications, energy production and mining, all of which could drive the taxon to Critically Endangered (CR) or Extinct (EX) in a very short time (IUCN 2020). Following Guidelines for IUCN Red List Categories and Criteria (2020), *H. flabellifera* should be categorised as Vulnerable (VU D2).

#### Phylogenetic analysis.

**Figure 4.**
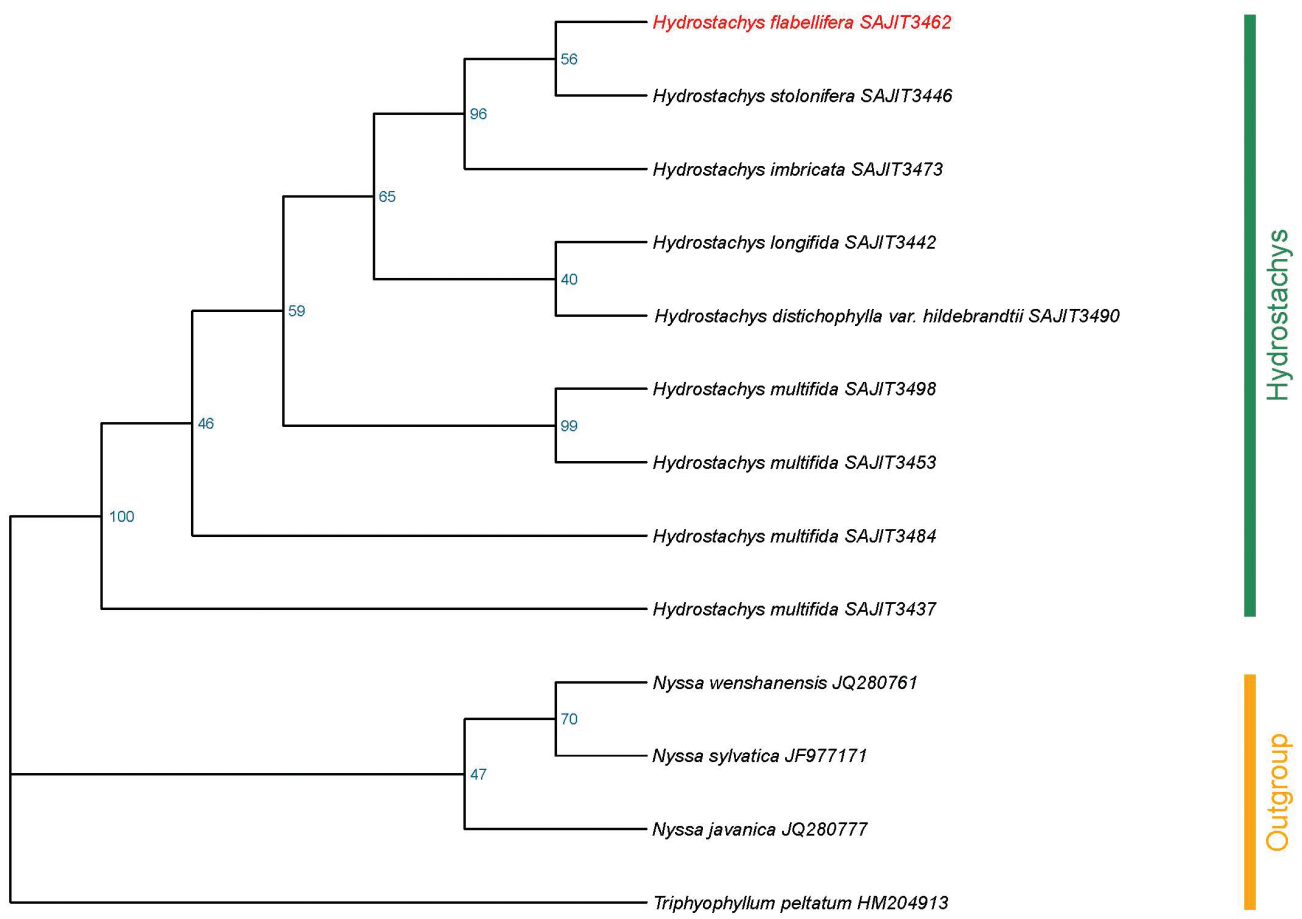
Maximum Likelihood tree, based on ITS. Bootstrap values are labelled alongside each node.

*Hydrostachys flabellifera* was placed in a robust clade together with *H. stolonifera* and *H. imbricata* (Fig. 4) with a high bootstrap support (BS = 96%), while they share limited morphological characteristics. *H. multifida*, considered as a clade in morphology, is not a monophyletic group, although with low bootstrap support.

## Discussion

*Hydrostachys* are mostly annual, only a limited number of species with stolons are perennial (Perrier 1952), like *H. monoica* and *H. stolonifera*. In this case, we did not observe any structure, like stolons, that could help *H. flabellifera* live for many more years. Therefore, *H. flabellifera* probably is annual, but continuous observation is needed.

*Hydrostachys flabellifera* closely resembles *H. verruculosa* and *H. laciniata* in having simple leaves, but can be easily recognised by several characters. The leaf emergences of the latter two species are densely arranged and overlapping and they constitute a thick cylindrical leaf with indistinct rachis. In contrast, the rachis of *H. flabellifera* are thin and obvious and the emergences stretch out into layers, sparsely arranged and significantly reduced at the lower leaves. Their segments patterns on male bract are also obviously different. *H. laciniata* was once treated as a form of *H. verruculosa* (Perrier 1952) after it was published as a new species (Warming 1899). Cusset (1973) also recognised it as a distinct species and further presented a diagram to compare it with *H. verruculosa* and that diagram clearly showed the differences in the bracts and the emergences on the leaf. After examining the type specimens of these two species, we accepted the treatment of Cusset (1973) and continued to compare the new species with these two species. Combining with morphology, phylogenetic results also provide solid evidence for the newly-discovered species. *Hydrostachys flabellifera*, *H. stolonifera* and *H. imbricata* form a robust clade, but share limited morphological characters. *H. stolonifera* and *H. imbricata* are much larger than *H. flabellifera* in size and they are definitely different in leaf types. H. distichophylla var. hildebrandtii is similar to *H. flabellifera* in morphology, but they are located in two distinct clades. The phylogenetic position of *H. flabellifera* would be much clearer when more taxa are included in the analysis. The combined results from phylogenetic analysis and detailed comparisons of morphological characteristics amongst *H. flabellifera*, *H. verruculosa*, *H. laciniata*, H. distichophylla var. distichophylla and *H. stolonifera* are listed in Table 2.

**Table 2.** Comparison of morphological characteristics of *Hydrostachys flabellifera*, *H. verruculosa*, *H. laciniata*, H. distichophylla var. distichophylla, and *H. stolonifera*.

Characters	*Hydrostachys flabellifera*	*H. verruculosa*	*H. laciniata*	H. distichophylla var. distichophylla	*H. stolonifera*
Leaf division	Simple	Simple	Simple	Simple	Tripinnatifid
Leaf length	3–12 cm	4–20 cm	10–30 cm	20–40 cm	2–7 cm
Leaf emergences arrangement	Sparsely arranged, not overlapped	Densely arranged, overlapped	Densely arranged, overlapped	Sparsely arranged, not overlapped	Sparsely arranged, not overlapped
Leaf emergence shape	Flabellate, palmately parted	Obovate, margin entire	Irregular shape with laciniate margin	Falcate, margin entire	Often falcate, margin entire
Appendix of leaf emergence	With cilia at the apex	Glabrous or with short cilia or tufts of cilia at the apex	Without cilia	Without cilia	Without cilia
Length of male spike (including peduncle)	5.4–8 cm	4–13 cm	5–10 cm	10–30 cm	1–8 cm
Male bract	Rhombic, 1–2.2 mm × 1–2.2 mm	Rhombic, 3 mm × 3 mm	Sub-rhombic, ca. 3 mm × 3 mm	Rounded, 1.6 mm in diameter	Rhombic, 2–2.5 mm × 2.5–3 mm
Segment I on male bract	Entire, margins sinuous, flanked by 1–2 tiny lobules on each side, apex acute	Entire, margins sinuous, flanked by 1–2 lobules on each side, apex obtuse	3-lobed, the medium lobe larger than the lateral ones, apex of lobes obtuse to rounded	Entire, apex rounded or slightly angular	Generally entire, sometimes lobulated laterally, apex acute, obtuse or rounded
Segments II on male bract	2 rows, each row with 3–5 separated lobules, ca. 0.3 mm high, apex acute or obtuse	2–3 rows, each row with 3–4 lobules, 0.6 mm high, apex angular or acute	2 rows, upper row with one larger lobule, lower row with 4–5 smaller lobules, 0.3–0.6 mm high, apex obtuse or rounded	Without segments II	Generally one row with 3 lobules, 0.7–0.8 mm high, apex rounded

*Hydrostachys* are adaptable to diverse aquatic environments, from clean mountain streams to muddy rivers. These species can be distinguished by the type of leaves, emergences, spikes, also the bract shape and segments arrangement are valuable identification characteristics. Due to different statuses between fresh plant and pressed specimens, greater attention to detail is needed when comparing and describing these species in different conditions. Based on our empirical research, in some specific cases, it is tricky to connect the living plant to the corresponding dry specimens. We highly recommend combining field investigations and herbarium examinations to obtain the full knowledge of this aquatic family.

### Key to identification of *Hydrostachys* in Madagascar

**Table:** 

1	Leaf simple	**2**
–	Leaf 1–4-pinnate	**5**
2	Cylindrical leaf with emergences densely arranged and overlapped	**3**
–	Emergences sparsely arranged and stretched out	**4**
3	Dorsal side of the female bract densely covered with emergences	***H. verruculosa***
–	Dorsal side of the female bract with bare surface, only few emergences on the top	***H. laciniata***
4	Emergences falcate, margin entire	**H. distichophylla var. distichophylla**
–	Emergences flabellate, palmately parted	***H. flabellifera***
5	Plants with stolons; leaf in indefinite growth	**6**
–	Plants without stolon; leaf in definite growth	**7**
6	Leaf pinnate or bipinnate, yellowish-white or pale green	***H. monoica***
–	Leaf tripinnate, moss green or dark moss green	***H. stolonifera***
7	Leaf only once pinnate	**8**
–	Leaf more than once pinnate	**11**
8	Pinnules bearing long and capillary emergences	***H. longifida***
–	Pinnules bearing scale-like emergences	**9**
9	Pinnules distantly arranged, terminated with a brush in the rainy season	**H. distichophylla var. hildebrandtii**
–	Pinnules closely arranged, without brush at the apex	**10**
10	Petiole bare at the base, upper part covered with short emergences	***H. plumosa***
–	Petiole completely covered with imbricata emergences	***H. imbricata***
11	The middle of the leaf wider than the base and the top	.. ***H. multifida***
–	The base of the leaf wider than the upper	**12**
12	Pinnule covered with capillary emergences	**13**
–	Pinnule covered with scale-like or irregular emergences	**14**
13	Leaf divided into 3–5 pinnae; petiole covered with small spatulate emergences	***H. trifaria***
–	Leaf divided into 5–20 pinnae; petiole without obvious emergences but bristles	***H. decaryi***
14	Leaf large, 3–4-pinnate; rhizome fist-sized; petiole 0.5–1 m long	***H. maxima***
–	Leaf short, 1–3-pinnate; rhizome smaller; petiole less than 0.1 m long	**15**
15	Leaf irregularly 1–2-pinnate; petiole and rachis covered with few distant emergences	***H. perrieri***
–	Leaf regularly 2–3-pinnate; petiole and rachis densely covered with emergences	***H. fimbriata***

## Supplementary Material

XML Treatment for Hydrostachys flabellifera
